# Supplementary material for: Rapid heat discharge during deep-sea eruptions generates megaplumes and disperses tephra
Source: Nat Commun. 2021 Apr 21;12:2292. doi: 10.1038/s41467-021-22439-y (PMC8060308; doi:10.1038/s41467-021-22439-y)
Supplement: Supplementary file 1 — Supplementary Information [file 41467_2021_22439_MOESM1_ESM.pdf]

SUPPLEMENTARY INFORMATION

Rapid heat discharge during deep-sea eruptions generates megaplumes and disperses tephra

Nature Communications, 2021

Samuel S. Pegler and David J. Ferguson

Supplementary Notes

Supplementary Note 1: Bounds on tidal dispersal

Continuous measurements from automated oceanic floats [1] show that tidal currents in the deep oceans form highly regular, symmetrical oscillations superposed with background noise, e.g. from eddies and internal waves. Due to the near-zero time average of tidal velocities over the course of a tidal cycle, both the settling of tephra and advection of the plume system will, under a purely tidal flow field, produce oscillations with a near-zero mean displacement. Hence, the total dispersal by tides is constrained to lie within a certain distance from the source representing the maximum distance a parcel is advected before the tide reverses. Material elements or particles released into a tidal field will undergo periodic, closed orbits in the vicinity of the vent. To show this, we compute the horizontal trajectories of fluid elements or particles, $[x(t), y(t)]$, advected under an illustrative tidal oscillation specified by

$$\dot{x} = U_x \cos\left(\omega t - \theta_x\right), \quad \dot{y} = U_y \cos(\omega t - \theta_y), \tag{22}$$

where U_x is the maximum tidal current in the zonal direction, U_y is the maximum tidal current in the meridional direction, ω is the frequency of the tidal oscillation, θ_x and θ_y are phase shifts, and the dot denotes a time derivative. Near the NESCA lava flow, the tidal currents reach magnitudes of approximately 5 cm s^{-1} [1]. To simulate an illustrative tidal pattern, we set $\omega = 4\pi/T$, where T is the duration of one day. Supplementary Figure 1A shows the dispersal pattern accumulated following the continuous composition of trajectories of parcels released continuously over the course of one day for an example: $U_x = 2$ cm s^{-1}, $U_y = 5$ cm s^{-1}, with $\theta_x = t_0\omega$ and $\theta_y = t_0\omega + \pi/2$. The pattern developed by tidal dispersal is confirmed to produce a closed region with an elliptical rim. To determine the rim position for general θ_x, θ_y, U_x and U_y, we integrate Eqn. (22) subject to the initial release position $(0, 0)$, giving

$$x = \frac{U_x}{\omega}\left[\sin(\omega t - \theta_x) + \sin\theta_x\right], \tag{23}$$

$$y = \frac{U_y}{\omega}\left[\sin(\omega t - \theta_y) + \sin\theta_y\right], \tag{24}$$

describing elliptical trajectories. The rim of the tidal dispersal pattern is the locus of maximum radii arising as t_0 is varied over one tidal cycle. The maximum distance of a particle released at $t = t_0$ occurs at $t = t_0 + \pi/\omega$. Substituting this value into (23) and (24), we determine the rim position parametrised as a function of t_0, given by

$$R(t_0) = \frac{2}{\omega}\left[U_x^2\cos^2(\omega t_0) + U_y^2\cos^2(\omega t_0 - \Delta\theta)\right]^{1/2}, \tag{25}$$

where $\theta_x = \omega t_0$ and $\Delta\theta = \theta_y - \omega t_0$. Since $0 \le \cos^2 \le 1$, it follows that the maximum dispersal distance is bounded by $L_{tide} = 2U/\omega = UT/2\pi$, where U is the larger of U_x and U_y. Mixed

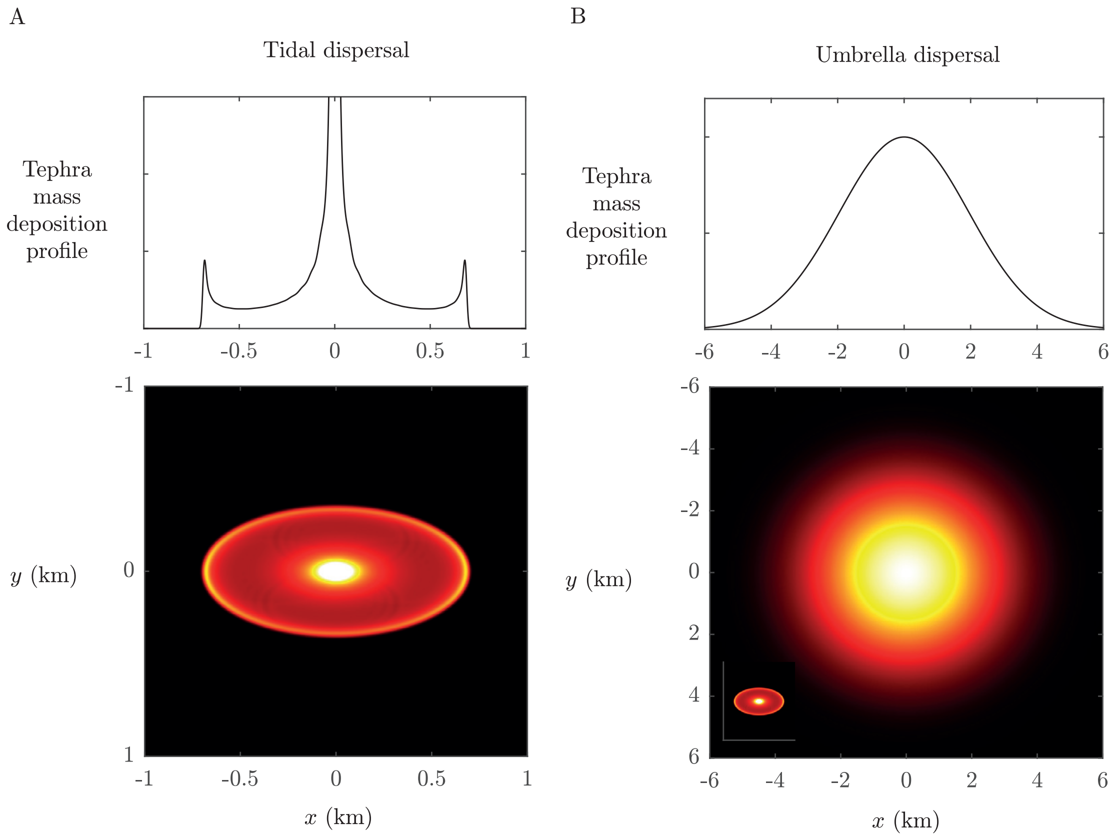

Supplementary Figure 1. Plan-views of the typical tephra deposition fields produced by tides or by buoyancy-driven lateral transport in the umbrella of the hydrothermal plume. A characteristic dispersal field from (A) tidal advection and (B) advection within a buoyancy-driven umbrella, representing two endmembers for submarine tephra dispersal. Blue represents no particles, while yellow represents maximal deposited concentration. The tidal pattern shown in A is given by superposing trajectories of particles released continuously over the course of one day predicted by the kinematic model of Eqn. (22) of Supplementary Note 1, showing the maximum dispersal distance of L_{tide} by tidal oscillation. For comparison, the buoyancy-driven pattern showing panel (B) is given by the prediction of Eqn. (1) of the paper. The case $L = 4.9$ km is illustrated, corresponding to the dispersal distance inferred by fitting the deposition profiles for Northern Escanaba (NESCA) (shown in Fig. 2 of the paper). Upper panels show cross-sections of the dispersal patterns, illustrating the fundamentally different decay characteristics and dispersal distances operating under the two mechanisms. Panel (B) shows the tidal dispersal on the same scale as an inset, illustrating its considerably smaller scale.

diurnal and semi-diurnal patterns can be modeled by superposition of two patterns of the form of Eqn. (22) above with one frequency twice the other. This would result in more complex patterns than purely elliptical, but are likewise constrained by the maximum tidal distance of L_{tide}, with U the maximum tidal speed in any given direction. For typical tidal currents in the NESCA region estimated from Argo floats [1], $U \lesssim 5$ cm s^{-1}, and hence $L_{tide} \lesssim 0.7$ km. In the absence of buoyancy-driven expansion of the umbrella, the independent effect of tides is thus to advect the plume-particle system back and forth within a radius of oscillation L_{tide}.

In addition to the closure of the orbit, a notable characteristic of purely tidal dispersal is the development of a sharp local maximum at the 'rim' of the tephra deposition pattern, evident in Supplementary Figure 1A (at least for a pure diurnal or semi-diurnal pattern; mixed tides, as apply in the NE Pacific, will produce a more complex pattern, though a similar constraint on the orbit will apply). This phenomenon is a consequence of the lingering of the trajectories near the rim as they switch direction during tidal reversal. Another characteristic is the non-axisymmetric eccentricity of the tidal dispersal pattern, which will generally arise for $U_x \neq U_y$ and $\theta_x \neq \theta_y$.

The deposition of tephra around the NESCA lava of more than 5 km in all directions is considerably larger than the maximum tidal dispersal scale $L_{tide} \sim 0.7$ km. In view of this, tidal advection cannot account for the observed deposition pattern at NESCA. We therefore propose that the dispersal was instead driven predominantly by buoyancy-driven radial flow of the umbrella along a neutral level, as detailed in the Methods. The predicted deposition due to this process is illustrated in Supplementary Figure 1B.

SUPPLEMENTARY NOTE 2: APPROXIMATING A POLYDISPERSE PARTICLE DISTRIBUTION USING A MONODISPERSE THEORY

In our analysis, we choose a representative settling speed w_s for the group of particles we consider ($d = 250$-500 μm) and apply the monodisperse theory represented by Eqn. (1) of the main paper. To understand the strength of this approximation in a given situation, we consider here a generalized theoretical description of particle deposition allowing for a polydisperse distribution and use it to determine the strength of approximation of assuming a representative settling speed for a subset of particle settling speeds. Since the concentration of particles is highly dilute (see Methods), the total mass of deposited tephra per unit area of the seafloor $\Omega(r)$ at a distance from the source r by the weighted integral superposition:

$$\Omega(r) = \int_0^\infty F_0(d) \mathrm{e}^{-\pi[r/L(d)]^2} \, \mathrm{d}d \equiv \int_0^\infty F(d,r) \, \mathrm{d}d, \tag{26}$$

where $F_0(d)$ is a weighting function (with units of mass per unit areas per unit particle size), $F(d,r)$ denotes the integrand, $L(d) = \sqrt{Q_{umb}/w_s(d)}$ is the dispersal lengthscale for the particle species of size d, $w_s(d)$ is the settling speed of the particle of size d (see Methods), and $F(d,r)$ is the density function (defined as the integrand).

In conducting our inversion for the umbrella flux Q_{umb}, we apply a monodisperse approximation to the particle range $250 < d < 500$ µm. The deposited mass per unit area for this group is represented by the truncation:

$$\Omega(r) = \int_{d_0-\delta d}^{d_0-\delta d} F_0(d) \mathrm{e}^{-\pi[r/L(d)]^2} \, \mathrm{d}d, \tag{27}$$

where $d_0 = 325$ µm is the central value and $\delta d = 125$ µm. By expanding the integrand about the central value, evaluating the integrals, and noting that the first order correction evaluates to zero, we obtain the approximation:

$$\Omega(r) \approx 2\delta d F(d_0, r) + \tfrac{1}{3}\delta d^3 F_{dd}(d_0, r) \tag{28}$$

$$\approx \Omega_0 \mathrm{e}^{-\pi[r/L(d_0)]} + O(\delta d^3), \tag{29}$$

where $\Omega_0 = 2\delta d f(d_0)$.

Supplementary References

[1] Ollitrault, M. & Rannou, J.-P. ANDRO: an argo-based deep displacement dataset. *Journal of Atmospheric and Oceanic Technology* **30**, 759 – 788 (2013).
